# Oh, My Gauze !!!- A rare case report of laparoscopic removal of an incidentally discovered gossypiboma during laparoscopic cholecystectomy

**DOI:** 10.1016/j.ijscr.2020.04.058

**Published:** 2020-05-16

**Authors:** Jitendra Sankpal, Mukund Tayade, Jai Rathore, Atish Parikh, Deepak Gadekar, Shaba Fathima S, Sushrut Sankpal

**Affiliations:** Grant Government Medical College & Sir, JJ Group of Hospitals, Mumbai, India

**Keywords:** Retained surgical sponge, Gossypiboma, Textiloma, Laparoscopic removal of gossypibomas

## Abstract

**Introduction:**

The work has been reported in line with the SCARE criteria. The most common retained foreign body during surgery is woven cotton surgical sponge, which includes both laparotomy pads and smaller sponges. Sponges are easily retained because of their ubiquitous use, relatively small size and when soaked in blood, sponges conform to and can be difficult to distinguish from surrounding tissues. The problem of retained surgical sponge is known as gossypiboma, and also as ‘textiloma’, ‘gauzoma’ or ‘muslinoma’. The number of preoperatively diagnosed cases treated by laparoscopic approach is rare in the literature and laparoscopic removal of incidentally detected gossypiboma with concomitant laparoscopic cholecystectomy is not yet reported in the literature.

**Presentation of case:**

In 40-year-old female with caesarean section 5 years ago, now during elective laparoscopic cholecystectomy and umbilical hernia repair, an incidentally detected Gossypiboma was encountered. The Gossypiboma was safely excised by laparoscopic technique followed by laparoscopic cholecystectomy and open hernia repair.

**Discussion:**

Postoperative complications following surgery are common and mostly unavoidable but some like the gossypiboma are infrequent and avoidable. Most of them are asymptomatic and present in the body for a long period of time. However, once discovered these foreign bodies must be removed for which laparoscopy proves to be a better approach. Most of these cases also are under-reported due to medicolegal implications.

**Conclusion:**

Gossypiboma is one of the preventable complications of surgery by the compliance of certain measures perioperatively. If detected incidentally, it can be safely managed laparoscopically with a shorter post-operative hospital stay and better cosmetic results.

## Highlights

• Gossypiboma, textiloma and cottonoid foreign bodies are discussed.
• Measures to avoid retaining surgical items are reviewed.

## 1 Introduction

The work has been reported in line with the SCARE criteria [1]. Gossypiboma defines a mass lesion shaped by foreign body reaction developed around sponge or such substances retained in the operative area. Such retained foreign bodies are found to occur in 1 in 3000 to 1 in 5000 cases and are mostly under-reported due to fear of medicolegal issues. The patients may remain asymptomatic for long period of time or may even present acutely with symptoms of inflammation and sepsis. Investigations like plain radiographs (X-rays), ultrasound (USG), computed tomography (CT) or magnetic resonance imaging (MRI) may be helpful in diagnosing the condition in cases where there is a high index of suspicion. As in our case scenario, these gossypiboma may be detected incidentally during other procedures involving the same cavity and need to be removed when discovered.

We report here a case who presented with an incidental finding during laparoscopic cholecystectomy and umbilical hernia repair with a history of caesarean section done 5 years ago at some other hospital. The number of preoperatively diagnosed cases treated by laparoscopic approach is rare in the literature and laparoscopic removal of incidentally detected gossypiboma with concomitant laparoscopic cholecystectomy is not yet reported in the literature.

## 2 Presentation of case

A forty-year-old female patient was admitted for intermittent abdominal pain since, 6 months. She had no history of fever with chills or vomiting or any other bowel complaints. On examination her vital parameters were within normal limits and her abdomen was soft, nontender with no palpable lump barring umbilical hernia with a defect of 3 cm × 3 cm, and a vertical infra umbilical scar of previous surgery over hypogastrium. Her blood investigations were within normal limits.

Patient only revealed history of undergoing a classical caesarean section 5 years ago. She underwent ultrasonography of the abdomen, six months back which showed cholelithiasis with umbilical hernia and her symptoms were attributed to the radiological findings. She underwent a repeat ultrasonography after admission and similar findings were noted. After due informed consent she was taken for concomitant laparoscopic cholecystectomy and intraperitoneal onlay mesh plus (IPOM plus) repair for umbilical hernia.

**Fig. 1:**
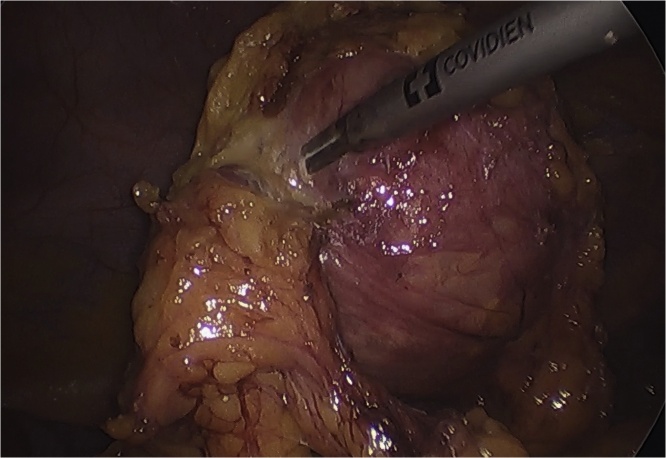
10 cm × 15 cm cystic mass as Gossypiboma.

Under general anesthesia with nasogastric tube and urinary catheter in place, patient position was reverse Trendelenburg with legs split and laparoscopy trolley near patient’s right shoulder. Since adhesions were anticipated in view of previous abdominal surgery and umbilical hernia, neither a technique of creating pneumoperitoneum by the Veress needle [2] nor the Hasson’s technique via trans-umbilical open laparoscopy [3] could be used, instead operating surgeon standing on left side of operation table, entry at the Palmer’s point [4] for creating pneumoperitoneum was used wherein the Veress needle was inserted 3 cm below the left costal margin in left upper quadrant in mid clavicular line and pneumoperitoneum was created and thereafter optical 5.5 mm trocar was placed at the Palmer’s point after removing the Veress needle. A 5 mm, 30-degree HD telescope was used for laparoscopy. On entering the abdomen, significant bunch of adhesions formed by greater omentum adhered along the lower midline scar and umbilical hernia with the greater omentum and small intestine being part of the hernial sac was found. First operating 5.5 mm trocar was inserted in left lumbar quadrant and upon careful adhesiolysis of greater omentum from the scar and reduction of contents under vision, from umbilical hernia, a single large cystic mass of 15 cm × 10 cm was found within the gastrocolic omentum which was abutting the stomach and transverse colon (Fig. 1).

Relatives of the patient were informed of the additional findings and consent for excision of mass was taken. Once scar was cleared off adhesions and contents of umbilical hernia was reduced, a second 10 mm operating trocar was inserted through the umbilicus and with use of ultrasonic energy devices, meticulous dissection of the mass was carried and once mass was excised out uneventfully from gastrocolic omentum, mass was parked temporarily over the stomach. Subsequently operating surgeon standing between split legs, telescope was moved to umbilical trocar and with standard third, 10 mm epigastric trocar, and fourth and fifth, 5.5 mm trocars at right mid clavicular and anterior axillary lines respectively uneventful laparoscopic cholecystectomy was completed (Fig. 3).

**Fig. 2:**
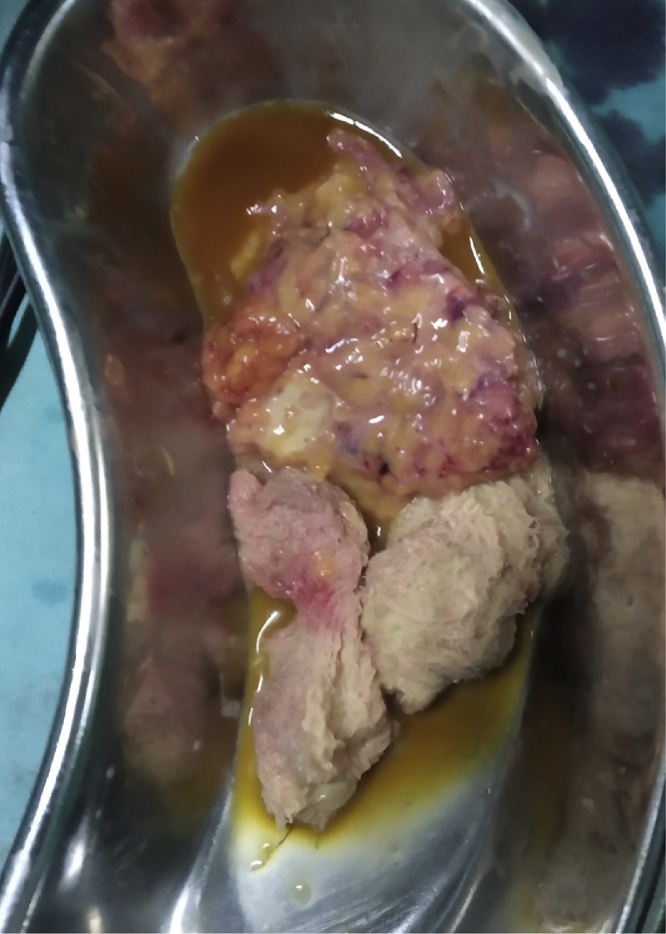
Cut open specimen of Gossypiboma with two surgical sponges and pus.

The gall bladder was removed first from the umbilical port following which the mass was placed in a endo bag for removal. However, during extraction of the bag there was accidental rupture of the cystic mass to reveal 70–80 ml of thick pus and two gauze sponges of size 10 × 10 cm each (Fig. 2). Remaining pus was suctioned out and the mass along with foreign bodies (gauze sponges) was extracted from the umbilical port successfully. A thorough wash of raw area in gastrocolic omentum was done with suction irrigation canula passed through Palmer’s point trocar [5]. Then 16 French intra-abdominal drain (naso-gastric tube) was inserted through right anterior axillary port and it was positioned in Morrison’s pouch. Instead of IPOM Plus repair, umbilical hernia was repaired using double breasting technique, with nonabsorbable polypropylene number 1 sutures since mesh was avoided in view of rupture of mass and possible pus contamination of umbilical port. All port sites were closed in regular fashion and patient was extubated uneventfully. The specimens were sent for histopathological and gauze sponges for forensic evaluation and pus was sent for culture respectively (Fig. 4). The histopathology of the cyst wall revealed changes of chronic foreign body granulomatous inflammation and pus culture was sterile. The post-operative recovery of the patient was uneventful. Her presenting symptom of pain in abdomen was abolished during follow up of two months.

**Fig. 3:**
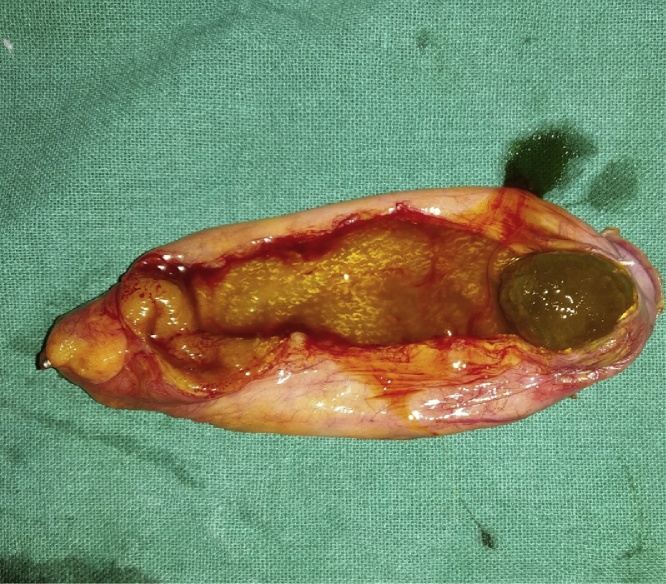
Laparoscopic Cholecystectomy specimen with gall stone.

**Fig. 4:**
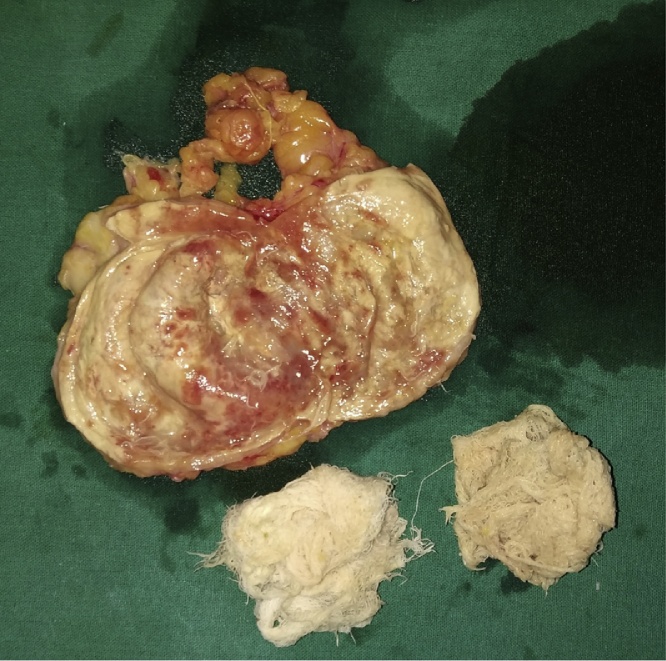
Cut section of Gossypiboma having 1 cm thick cyst wall with two surgical gauze sponges.

## 3 Discussion

The retained foreign body is a rare occurrence in surgery and was first described by Wilson et al in 1884 [6]. In a study conducted by Gawande et al. in 2003 in the U.S, the incidence was found to be of 1 in 8801 to 1 in 18,760 inpatient operations corresponding to one case or more each year for a typical large hospital [7]. The rates are more in cases involving the open cavities. Although no body cavity is spared from the occurrence of a retained surgical foreign body, the abdomen and the thorax are the most frequent sites in which they are found (74% and 11%, respectively) [8]. Gossypiboma accounts for 69% of surgical foreign bodies, whereas in remaining cases, it is a surgical instrument such as forceps, electrodes, or retractors [7]. The word, gossypiboma, derives from two sources: the Latin word “Gossypium” meaning textile or cotton, and the Swahili word “boma” meaning place of concealment. Other terms used include textiloma and gauzoma.

Gawande et al. identified three risk factors for retained sponges: emergency procedures, unplanned change in the procedure, and the patient’s body mass index [9]. It is more commonly seen in female sex in gynecologic operations since the width of the surgical site and difficulty of exploration increase the risk of oversight of the sponges for obese patients [10].

The retained foreign body causes two types of foreign body reactions. In the first one, adhesion, encapsulation, and granulation take place with an aseptic fibrotic reaction around the foreign body. In such cases the patient may not have apparent complaints and findings and it can be detected incidentally, as in our case. The latter one is the exudative reaction which causes cyst and abscess formations. In our case, approximately 1 cm thick capsule developed with pus formation around the retained laparotomy gauze pads during 5 years and it stayed asymptomatic [5]. The retained foreign body may transmurally immigrate to stomach, duodenum, small intestine, large intestine, diaphragm, bladder, and vagina. After migration the patients either may be asymptomatic or can present with perforation, obstruction, bleeding, or fistula findings [[11], [12], [13], [14]]. In our case, the patient revealed subsequently history of one miscarriage 6 months back, which may be attributed to gossypiboma. Therefore, diagnosis may be delayed and may cause serious morbidity and even mortality. A patient with previous abdominal surgery who presents later with abdominal pains, nausea, vomiting, and features of intestinal obstruction or malabsorption syndrome should be suspected to have abdominal gossypiboma.

It is difficult to diagnose retained items in asymptomatic patients. In the immediate phase, a plain radiograph may show evidence of the radio-opaque line on all modern sponges. However, up to 10% of these have been found to be falsely negative [15]. If seen in the delayed acute phase, they may present as a heterogenous mass, surrounded by fibrotic capsule, and containing gas. A presumptive diagnosis of a retained sponge should be further investigated with either ultrasound, CT, or MRI. A retained sponge is echogenic on ultrasound, and presents as a sharply delineated acoustic shadow, or a hypoechoic mass with a complex cystic pattern. CT reports suggest that a spongiform pattern, surrounded by a dense, enhancing rim is suggestive of a retained swab. If there remains any question an MRI can be performed [16]. However, the confirmed diagnosis is established only on exploration for which laparoscopy stands out to be a good option as in our case, the mass was found incidentally at the time of laparoscopic cholecystectomy.

Once gossypiboma is diagnosed, it can be removed both with open or laparoscopic surgery. A foreign body transmurally migrated to stomach or colon can be removed by endoscopy [17]. Laparoscopy has the advantages of shorter hospitalization, less postoperative pain, and better cosmetic appearance. There are limited number of studies with laparoscopic approach in the gossypiboma treatment [18]. Furthermore, laparotomy removal of Gossypibomas is not always feasible at the site of the previous operation scar, especially if it is successive to a Pfannenstiel incision, because of the frequent migration of the foreign body over the years [19]. In the reported case, a slightly different port placement anticipating the possibility of intra-abdominal adhesions, made the procedure easier and less time consuming, resulting in a better functional and cosmetic outcome.

Maintaining standard protocols helps to reduce this preventable complication of surgery. It can be decreased by keeping a thorough count at least thrice (preoperative, intraoperative and postoperative), especially during emergency operation, complete exploration of abdominal cavity by the surgeon before closure if there is any doubt in the counts. As per WHO recommendation, count should always be done separately in a consistent sequence by two similar persons with their name being noted in the count sheet or nursing record. Methodical exploration of surgical wound by the operating surgeon decreases the likelihood of leaving sponges [20]. If still there is a doubt regarding the gauze/mop count, immediate intraoperative X-ray should be done to detect gauzes if labelled with radio-opaque markers. Nowadays, newer modalities like two-dimensional bar code, radiofrequency detector, and radiofrequency identification are used where facilities are available for the same. Retained foreign body in abdomen, detected immediately in post-operative period or few months/years later should be removed as soon as possible, with patient and their relatives kept in the loop, whatever may be further consequences for operating team and hospital management.

## 4 Conclusion

Gossypiboma may be a life-threatening problem and is an avoidable complication of previous surgery. This differential should be always borne in mind while dealing with a patient with or without abdominal symptoms with previous operative history. Furthermore, the discovery of a gossypiboma can also lead to serious consequences for the surgeon involved in terms of a potential medicolegal challenge, and from criticism both publicly and within the medical profession.

Once detected the retained gauze pieces should be removed by surgery. During laparoscopic surgery for any other pathology, if incidental gossypiboma are detected, same can be excised laparoscopically with the advantages of smaller incision, less pain, shorter hospitalization, reduced hemorrhage and infection rates compared to open surgery. Thus, laparoscopy can be an excellent method and also the method of choice for retrieval of such incidental foreign bodies. But it should not be forgotten that prevention is always better than cure.

## Declaration of Competing Interest

No conflict of interest with all authors/nothing to disclose for all authors.

## Sources of funding

No funding.

## Ethical approval

Ethical clearance not taken since it is an single case report. Patient consent obtained.

## Consent

Written informed consent was obtained from the patient for publication of this case report and accompanying images. A copy of the written consent is available for review by the Editor-in-Chief of this journal on request”.

## Author contribution

1. Dr. Jitendra Sankpal – Corresponding Author and Chief Operating Surgeon.
2. Dr Mukund Tayade. Assisted Surgery.
3. Dr. Jai Rathore – Assisted Surgery.
4. Dr. Atish Parikh – Assisted Surgery.
5. Dr. Deepak Gadekar – Assisted Surgery.
6. Dr. S Shaba Fathima – Assisted Surgery.
7. Dr. Sushrut Sankpal – Writing Paper & study design.

## Registration of research studies

No research involved.

## Guarantor

DR Jitendra Sankpal. MS, FACS, FICS, FMAS, FIAGES, FALS, FBMS.
